# Obese melanocortin-4 receptor-deficient rats exhibit augmented angiogenic balance and vasorelaxation during pregnancy

**DOI:** 10.1002/phy2.81

**Published:** 2013-09-10

**Authors:** Frank T Spradley, Ana C Palei, Joey P Granger

**Affiliations:** Department of Physiology and Biophysics, Cardiovascular-Renal Research Center, University of Mississippi Medical CenterJackson, Mississippi, 39216

**Keywords:** Angiogenic balance, blood pressure, MC4R, obesity, pregnancy, vasorelaxation

## Abstract

While obesity is a major risk factor for preeclampsia, the mechanisms linking obesity and hypertension during preeclampsia remain unclear. Hypertension in preeclampsia is associated with placental ischemia-induced release of antiangiogenic soluble fms-like tyrosine kinase (sFlt-1) into the maternal circulation, which antagonizes vascular endothelial growth factor (VEGF) promoting endothelial dysfunction. Haploinsufficiency, defined as loss of one copy of a gene via a mutation, of the melanocortin-4 receptor (MC4R) is the most common cause of monogenetic obesity in humans. The purpose of our study was to determine the effects of genetic obesity on angiogenic balance, endothelial function, and blood pressure in pregnant MC4R+/− and MC4R+/+ rats. At gestational day (GD) 18, body weight and total body fat mass were greater in MC4R+/− than MC4R+/+ rats. On GD 19, plasma sFlt-1 was not significantly different between groups. Interestingly, circulating VEGF was greater in the obese rats with the source being adipose tissue and not the placenta. Wire myography showed in third-order mesenteric arteries that sensitivity (logEC50) to endothelial-dependent and nitric oxide donor-induced vasorelaxation was greater in MC4R+/− versus MC4R+/+. Mean arterial blood pressure was similar between groups. In conclusion, under normal pregnant conditions, genetically obese pregnant animals have greater angiogenic balance and dependency of vasorelaxation on nitric oxide signaling protecting against the development of hypertension. However, we speculate that, in the face of reduced uterine perfusion, a rise in circulating placental factors that target and reduce nitric oxide bioavailability exposes the susceptibility of genetically obese animals to greater hypertension in pregnancy.

## Introduction

Preeclampsia is a pregnancy-specific disorder identified by hypertension and proteinuria occurring on or after the 20th week of gestation (Espinoza 2012). This disease is the number one obstetric complication of pregnancy in the United States (Clark et al. 2008) and developing countries (Osungbade and Ige 2011). The World Health Organization reports that worldwide 800 women per day die from pregnancy complications (World Health Organization 2012). The etiology of this disease is still unclear although there is growing evidence that placental ischemia plays a central role. Indicative of placental ischemia is the hypoxia-induced release of placental-derived factors into the maternal circulation such as the antiangiogenic soluble fms-like tyrosine kinase (sFlt-1). The pathophysiological impact of elevated sFlt-1 or angiogenic imbalance in pregnancy is a result of functional antagonism of vascular endothelial growth factor (VEGF) (Kendall and Thomas 1993; Cindrova-Davies et al. 2011). In normal pregnancy, the rise in VEGF is compulsory for the marked peripheral vasorelaxation of healthy gravidity (Geva et al. 2002; Hewitt et al. 2006). As expected, sFlt-1 infusion into normal pregnant rats and mice elicits hypertension along with aberrations of vasorelaxation identified by endothelial dysfunction and diminished smooth muscle responsiveness to nitric oxide (Maynard et al. 2003; Bridges et al. 2009; Murphy et al. 2010, 2012; Li et al. 2012). Collectively, these data highlight that angiogenic imbalance (reduced VEGF:sFlt-1 ratio) is especially deleterious to the maternal cardiovascular system.

The prevalence of preeclampsia is increasing worldwide and the U.S. is not exempt (Wallis et al. 2008; Osungbade and Ige 2011). This bolsters the importance of studying risk factors for this maternal disease. Intriguingly, the prevalence of preeclampsia is highest in the southeastern U.S. accompanying the occurrence of obesity of this region (Wallis et al. 2008; Akil and Ahmad 2011). A wealth of epidemiological evidence supports that obesity heightens preeclampsia rate (Bodnar et al. 2005a,b, 2007; Mbah et al. 2010; Roberts et al. 2011). In fact, increasing obesity, defined as a body mass index (BMI; kg/m^2^) of greater than or equal to 30, predicts an increased rate of the disease with massively obese pregnant women (BMI ≥50) presenting with four times the risk for preeclampsia compared to pregnant women in the normal weight range (BMI = 18.5–24.9) (Mbah et al. 2010). In spite of this evidence that obesity is a major risk factor for preeclampsia, mechanistic studies explaining this relationship are very limited.

The melanocortin-4 receptor (MC4R) is important for control of food intake, and loss of this receptor elicits obesity in laboratory animals (Chagnon et al. 1997; Huszar et al. 1997; Yeo et al. 1998; Mul et al. 2012). In humans, haploinsufficiency, defined as the loss of one copy of a gene due to mutation, of the MC4R is the most common cause of monogenic obesity (Farooqi et al. 2000; Vaisse et al. 2000; Lubrano-Berthelier et al. 2006; Calton et al. 2009). The MC4R+/− rat was developed by mutating this gene, which prevents the insertion of the receptors into the plasma membrane of cells (Mul et al. 2012). The purpose of the present study was to examine angiogenic balance, endothelial function, and blood pressure regulation in genetically obese MC4R+/− pregnant rats versus control MC4R+/+ pregnant rats.

## Materials and Methods

### Animals

All animal experiments were in accordance with the National Institutes of Health *Guide for the Care and Use of Laboratory Animals* with all animal protocols approved by the University of Mississippi Medical Center's Institutional Animal Care and Use Committee. Timed-pregnant melanocortin-4-receptor heterozygous (MC4R+/−) and wild-type wistar hannover (MC4R+/+) female rats were purchased from Transposagen Biopharmaceuticals, Inc. (Lexington, KY). Rats were maintained on NIH31 standard chow diet (#7013, unautoclaved; Harlan Laboratories, Indianapolis, IN) and water ad libitum. Rats were maintained on a 12:12 h light:dark cycle at 23°C. Additional timed-pregnant rats were generated at our institution. For this, virgin rats were purchased from the same company, and at 15 weeks old, timed-pregnant rats were generated using the following strategy: MC4R+/− females were mated with MC4R−/− males and MC4R+/+ females mated with MC4R+/+ males. The observation of sperm in vaginal smears was indicative of gestational day (GD) 0. The total number of timed-pregnant rats in the MC4R+/+ and MC4R+/− groups was 7–8. Body weight, total body fat mass, and total lean mass were assessed at GD 18 using an Echo-MRI-700 (Echo Medical Systems, Houston, TX). On the morning of GD 19, animals were fasted at ∼3 h prior to tissue harvest. On average, rats were 17 weeks old at GD 19.

### Mean arterial blood pressure measurements

On GD 18, under isoflurane anesthesia (Butler Schein Animal Health, Dublin, OH), indwelling catheters were implanted in the left carotid artery and exposed at the nape of the neck. Catheters consisted of V/1 tubing attached to V/3 tubing (Scientific Commondities Inc., Lake Havasu City, AZ). Approximately 2.5 cm of the V/3 end of the catheter was inserted into the carotid. Catheters were filled with sterile heparin-saline solution (300 mg/mL; Pfizer, New York City, NY) and stoppered with nail to maintain patency. On GD 19, rats were placed in restraint cages and catheters connected to pressure transducers (MLT0699, ADInstruments, Colorado Springs, CO) coupled to a computerized data acquisition system (PowerLab, ADInstruments). Animals were allowed to acclimate to restraint for approximately 1 h. Once hemodynamic readings stabilized, mean arterial blood pressure data were collected.

### Tissue harvest

On GD 19, under isoflurane anesthesia, a midline incision was made and uterine horns with fetuses exteriorized. Blood was collected from the abdominal aorta into Corvac separator tubes (Tyco Healthcare Kendall, Mansfield, MA) and into Vacutainer K_2_EDTA tubes (BD, Franklin Lakes, NJ). The number of viable and reabsorbed fetuses in each animal was recorded along with individual fetus and placenta weights. Heart, kidneys, spleen, visceral white adipose tissue (combined retroperitoneal fat mass and fat pads surrounding the uterus and bladder), and interscapular brown adipose tissue were weighed. Tissues were snap-frozen in liquid N_2_ until processed for biochemistry as detailed below. The mesenteric vascular arcade was isolated and placed in ice-cold physiological saline solution (PSS) for vasorelaxation studies as detailed below.

### Tissue homogenization

Whole placentas and retroperitoneal adipose tissue (100–150 mg) were homogenized by a FastPrep-24 Instrument for 2 min in 2 mL Lysing Matrix D Tubes (MP Biomedicals, Santa Ana, CA). For this, placentas were homogenized in 1 mL radioimmunoprecipitation buffer with a protease inhibitor cocktail and phenylmethylsulfonylfluoride and sodium orthovanadate per manufacturer's instructions (Santa Cruz Biotechnology, Santa Cruz, CA). Adipose tissue was homogenized in 1 mL lysis buffer consisting of 1 mmol/L EDTA tetrasodium salt dihydrate (Fisher Scientific, Waltham, MA), 10 mmol/L Tris (pH 7.5), and 0.25 mol/L sucrose along with 0.6 mmol/L phenylmethylsulfonylfluoride, 1 mmol/L sodium orthovanadate, 0.27 U/mL aprotinin, 10 μL/mL protease inhibitor cocktail for mammalian cells, and 10 μL/mL phosphatase inhibitor cocktail 2 (Sigma, St. Louis, MO). Homogenates were quickly centrifuged at room temperature at 13,200 rpm. Supernatants were stored at −80°C until analyzed. Total protein concentration in each sample was quantified by the BCA method (Thermo Scientific, Rockford, IL).

### Biochemistry

Placental and adipose tissue sFlt-1, VEGF, and tumor necrosis factor (TNF) alpha levels were quantified by enzyme-linked immunosorbent assay (ELISA) per manufacturer's instructions (R&D Systems, Minneapolis, MS) and normalized to total protein. Plasma levels of VEGF (R&D Systems) was examined by ELISA and glucose (Cayman Chemicals, Ann Arbor, MI), total cholesterol (Wako Diagnostics, Osaka, Japan), free fatty acids (Zen-bio, Durham, NC), and triglycerides (Cayman Chemicals) were assessed by colorimetric assays. Circulating TNF alpha, leptin, adiponectin were examined in serum by ELISA (R&D Systems).

### Vasorelaxation studies

Third-order mesenteric arteries were cleaned of perivascular adipose tissue for vascular function studies as described previously (Spradley et al. 2012). Vascular rings of approximately 2.5 mm in length were mounted on chucks in a wire myograph (model 620M, Danish Myo Technology A/S, Aarhus, Denmark) containing 5 mL PSS (concentration in mmol/L: 118.3 NaCl, 4.7 KCl, 2.5 CaCl_2_, 1.2 MgSO_4_, 1.2 KH_2_PO_4_, 25 NaHCO_3_, and 11.1 dextrose) (Sigma) warmed to 37°C and bubbled with 95%O_2_/5%CO_2_. Four mN of preload was placed on the arterial rings. Blood vessel integrity was examined with a bolus dose of phenylephrine (PE) to produce vasoconstriction followed by a bolus of acetylcholine (ACh) to produce vasorelaxation, ensuring proper endothelial function. Only those arterial segments that reached 100% relaxation were included in our myography studies. Arterial segments were washed with PSS, equilibrated for 15 min, constricted with PE (∼10 μmol/L), and cumulative concentration–response curves generated to ACh [1E-18M to 3E-7M] (Sigma). After segments were washed, equilibrated, and constricted again, response curves were generated to sodium nitroprusside (SNP) [1E-10 to 3E-5] (Sigma).

### Statistical analysis

Data were graphed and analyzed (one-tailed *t*-test) using GraphPad Prism (La Jolla, CA). The logEC50 values in the myography experiments were determined by GraphPad Prism. The only goodness-of-fit criterion used for these values was, if the values were negative, they were to be removed; we had no negative values. Data are expressed as mean ± SEM. *P* < 0.05 was considered statistically significant.

## Results

### Body weights in obese MC4R+/− rats

**Figure 1:**
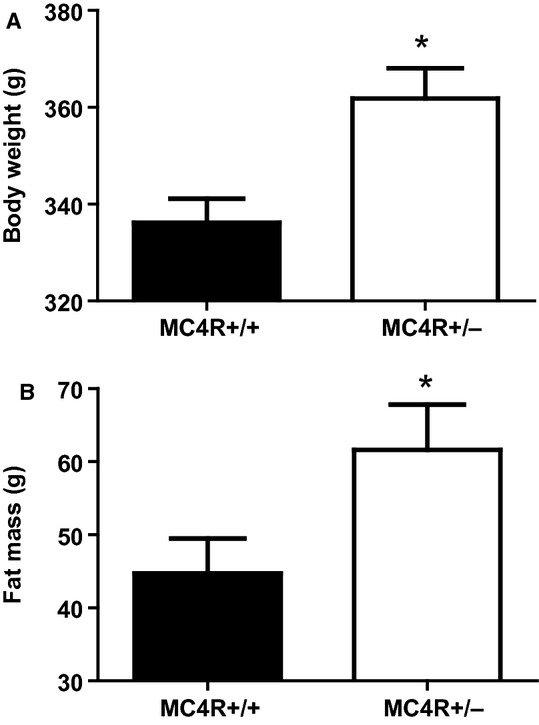
Body weight (A) and total body fat mass (B) in lean MC4R+/+ and obese MC4R+/− pregnant rats at GD 18. *P < 0.05 versus MC4R+/+.

At GD 18, body weight (Fig. 1A) and total fat mass (Fig. 1B) were significantly greater in MC4R+/− compared to MC4R+/+ rats. Lean mass was also greater in the MC4R+/− group although tibia lengths were similar between obese and lean groups (Table 1). At GD 19, visceral white adipose tissue mass (Table 1) was numerically greater in MC4R+/− whereas interscapular brown adipose tissue mass was similar between obese and lean pregnant rats (Table 1). Kidney weights were greater in obese pregnant rats, however, the weights of hearts, adrenals, and spleens were similar between groups (Table 1).

**Table 1:** Physical and circulating metabolic parameters of pregnancies at gestational day (GD) 19

Parameter	MC4R+/+	MC4R+/−
Lean mass (g)	255 ± 4	271 ± 4*
Visceral white adipose tissue (g)	14 ± 2	18 ± 2
Interscapular brown fat (g)	0.7 ± 0.1	0.8 ± 0.1
Tibia length (cm)	3.37 ± 0.02	3.43 ± 0.03
Number of fetuses	12 ± 1	12 ± 1
Reabsorbed fetuses	0 ± 0	0.5 ± 0.2*
Heart (g)	0.80 ± 0.05	0.90 ± 0.04
Kidneys (g)	1.58 ± 0.04	1.80 ± 0.03*
Adrenals (g)	0.06 ± 0.01	0.08 ± 0.02
Spleen (g)	0.53 ± 0.03	0.54 ± 0.04
Total cholesterol (mg/dL)	199 ± 24	298 ± 32*
Triglycerides (mg/dL)	475 ± 81	442 ± 70
Free fatty acids (μmol/L)	7250 ± 2420	9205 ± 2624
Glucose (mg/dL)	208 ± 9	242 ± 29
Insulin (ng/mL)	0.60 ± 0.07	0.64 ± 0.06
Leptin (ng/mL)	3.38 ± 0.63	5.03 ± 1.07
Adiponectin (ng/mL)	11.94 ± 1.53	9.57 ± 0.51

* *P* < 0.05 versus MC4R+/+ pregnant rats.

**Figure 2:**
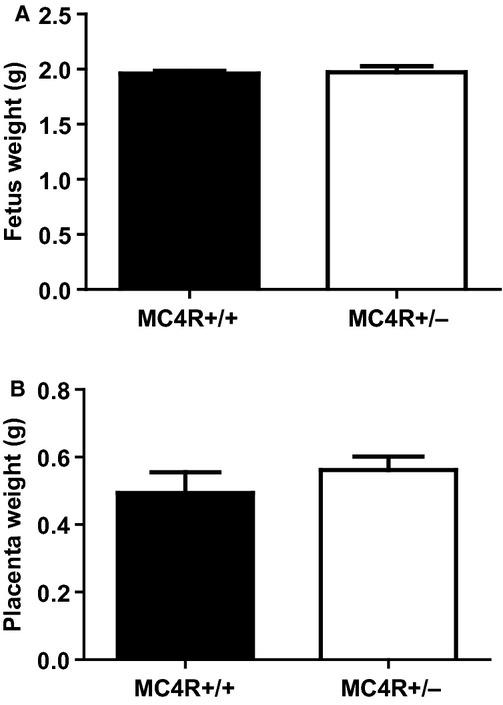
Fetus weights (A) and placenta weights (B) in lean MC4R+/+ and obese MC4R+/− pregnant rats at GD 19.

GD 19 fetal and placental weights were similar between obese MC4R+/− and lean MC4R+/+ pregnant rats (Fig. 2A and B, respectively). The number of live fetuses was similar between groups although there was a slight but significant increase in number of fetuses that were reabsorbed in MC4R+/− dams (Table 1).

### Tissue and circulating angiogenic factors in obese MC4R+/− rats

**Figure 3:**
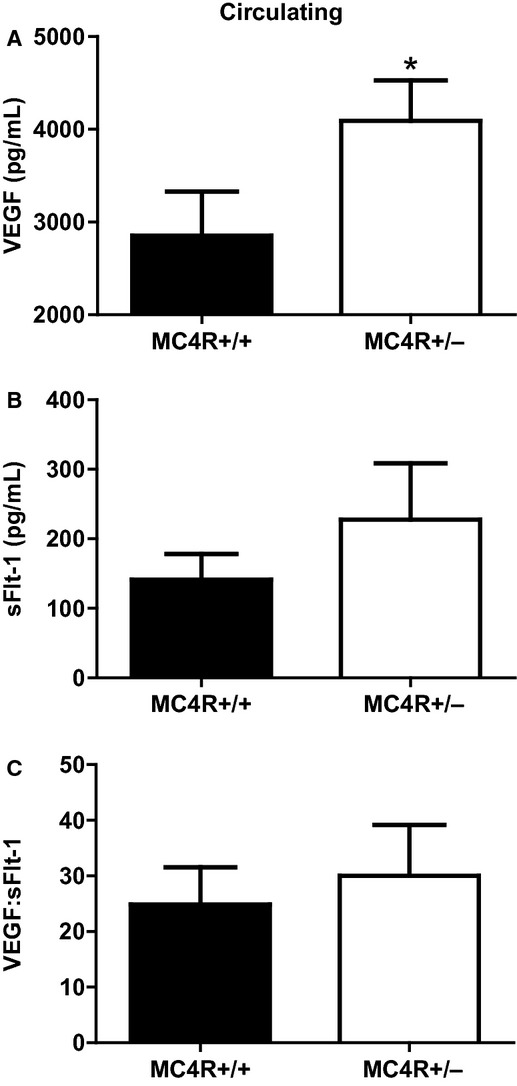
Circulating levels of VEGF (A), sFlt-1 (B), and the VEGF:sFlt-1 ratio (C) in lean MC4R+/+ and obese MC4R+/− pregnant rats at GD 19. *P < 0.05 versus MC4R+/+.

Circulating VEGF was significantly greater in obese MC4R+/− versus lean MC4R+/+ pregnant rats (Fig. 3A) whereas circulating sFlt-1 was marginally increased in obese pregnant rats (Fig. 3B) at GD 19. The circulating VEGF:sFlt-1 ratio, a marker of angiogenic balance, was similar between obese and lean pregnant rats (Fig. 3C).

**Figure 4:**
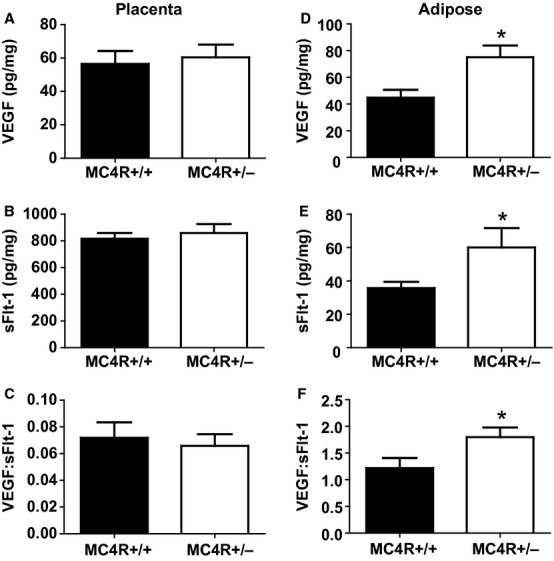
Placental levels of VEGF (A), sFlt-1 (B), and the VEGF:sFlt-1 ratio (C) and retroperitoneal adipose tissue levels of VEGF (D), sFlt-1 (E), and the VEGF:sFlt-1 ratio (F) in lean MC4R+/+ and obese MC4R+/− pregnant rats at GD 19. *P < 0.05 versus MC4R+/+.

To determine the source of the increased circulating VEGF in obese pregnant rats, placental angiogenic balance was assessed. However, VEGF (Fig. 4A), sFlt-1 (Fig. 4B), and the VEGF:sFlt-1 ratio (Fig. 4C) were similar between obese MC4R+/− and lean MC4R+/+ pregnant rats. Most interestingly, retroperitoneal fat from obese pregnant rats expressed significantly greater VEGF (Fig. 4D), sFlt-1 (Fig. 4E), and VEGF:sFlt-1 ratio (Fig. 4F).

### Vascular function in obese MC4R+/− rats

**Figure 5:**
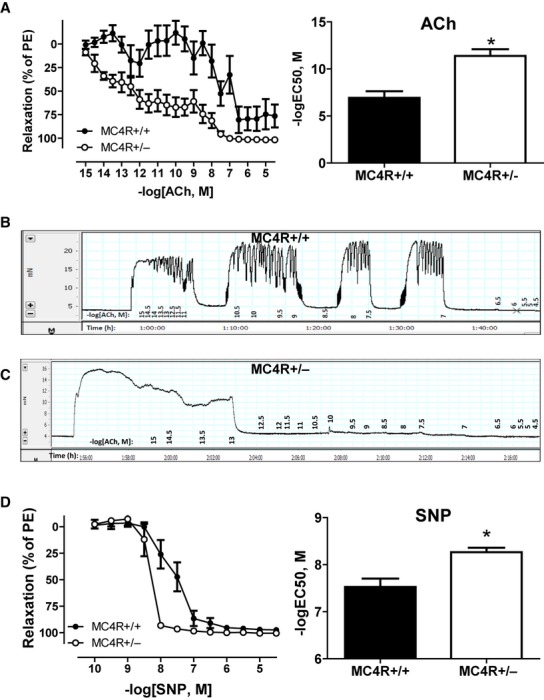
Cumulative concentration–response curves (left) and logEC50 (right) to endothelial-dependent vasorelaxation to acetylcholine (ACh) (A) in third-order mesenteric arteries from lean MC4R+/+ and obese MC4R+/− pregnant rats at GD 19. Raw tracings to ACh in MC4R+/+ (B) and MC4R+/− (C). Cumulative concentration–response curves (left) and logEC50 (right) to the nitric oxide donor sodium nitroprusside (SNP) (D). *P < 0.05 versus MC4R+/+.

Endothelial-dependent vasorelaxation was greater in mesenteric arteries from obese pregnant MC4R+/− with a noticeable reduction in sensitivity (logEC50) to acetylcholine compared to those from lean pregnant MC4R+/+ counterparts (Fig. 5A–C). Similarly, logEC50 response to endothelial-independent vasorelaxation to sodium nitroprusside was more pronounced in the obese pregnant rats (Fig. 5D).

### Blood pressure in obese MC4R+/− rats

**Figure 6:**
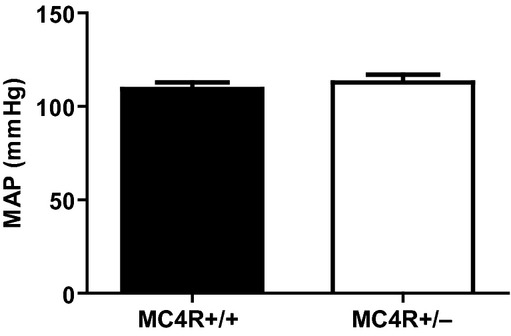
Mean arterial blood pressure (MAP) in lean MC4R+/+ and obese MC4R+/− pregnant rats at GD 19.

Figure 6 illustrates that mean arterial blood pressure was similar between obese MC4R+/− and lean MC4R+/+ pregnant dams.

### Circulating metabolic and adipokine profile in obese MC4R+/− rats

On GD 19, circulating total cholesterol levels were greater in obese MC4R+/− over lean MC4R+/+ pregnant rats whereas triglycerides and free fatty acids were similar between groups (Table 1). Glucose and insulin levels were also similar between groups (Table 1). Although not statistically significant, leptin was elevated 1.5 times in obese rats over that of lean rats without any difference in adiponectin levels between groups (Table 1). Circulating TNF alpha was greater in obese versus lean pregnant rats (25 ± 3 vs. 15 ± 4 pg/mL; *P* < 0.05, *N* = 6–7). However, there was no difference in TNF alpha between placental (2 ± 0.2 vs. 2 ± 0.2 pg/mg) and retroperitoneal adipose tissue levels (6 ± 0.9 vs. 6 ± 0.7 pg/mg) when, respectively, comparing obese and lean pregnant dams.

### Prepregnancy weights in obese MC4R+/− rats

Prepregnancy body weight (298 ± 6 vs. 267 ± 3 g), total body fat mass (56 ± 3 vs. 44 ± 1 g), total body lean mass (221 ± 5 vs. 206 ± 2 g), and food intake (129.5 ± 0.3 vs. 102.3 ± 4.1 g/week) were greater (*P* < 0.05, *N* = 4) in MC4R+/− versus MC4R+/+ rats, respectively, at 15 weeks old.

## Discussion

In the present study, we report that pregnant MC4R+/− rats were obese with greater fat mass compared to wild type MC4R+/+ pregnant rats. Interestingly, these obese pregnant rats presented with a proangiogenic phenotype. While circulating VEGF levels were greater in MC4R+/− versus MC4R+/+ pregnant rats, the antiangiogenic factor sFlt-1 was not significantly different between groups. Intriguing was the finding that the placenta was not the source of the elevated circulating VEGF levels found in the obese pregnant rats. However, when white adipose tissue was processed, it was found to have an exaggerated angiogenic balance represented by a greater VEGF:sFlt-1 ratio. Additional vasoprotective mechanisms were observed in the obese pregnant rats, which had greater vasorelaxation responses to endothelial-dependent and an exogenous nitric oxide donor. Moreover, the proinflammatory factor TNF alpha was increased in the circulation of obese versus lean pregnant rats. These studies were conducted under normal pregnant conditions where we found that obese MC4R+/− pregnant rats were protected against hypertension.

The motivation for initiating these studies is the knowledge that obesity is linked to increased preeclampsia rate (Bodnar et al. 2005a,b, 2007; Mbah et al. 2010; Roberts et al. 2011). Epidemiological studies have shown this relationship mainly by using BMI to identify obese gravid women (prepregnancy BMI >30). A striking report by Mbah et al. showed a stepwise increase in preeclampsia rate with increasing BMI. Those individuals having super obesity (prepregnancy BMI ≥50) had four times greater rate of preeclampsia (Mbah et al. 2010). Far fewer studies have assessed the mechanisms of this phenomenon. Attempts to address this were carried out in high-fat diet-induced obese rodents. Sprague Dawley rats maintained from 21 days old on a 16-week high-fat diet prior to pregnancy had increased body weight and systolic blood pressure, as measured tail cuff, reduced pup weight, and placental hypoxia at GD 15 (Hayes et al. 2012). While these data support that high-fat diet elicits preeclampsia symptoms, warranted were studies characterizing cardiovascular outcomes of pregnancy in a genetically obese model, which would be independent of a high-fat diet. The most common origin of monogenetic obesity in humans is deficiency, more specifically haploinsufficiency (where a single copy of a gene is inactivated by a mutation), of the MC4R (Farooqi et al. 2000; Vaisse et al. 2000; Lubrano-Berthelier et al. 2006; Calton et al. 2009). This receptor is important for body weight homeostasis. Rodents lacking this receptor are obese due to hyperphagia (Huszar et al. 1997; Mul et al. 2012). As expected, MC4R+/− nonpregnant rats presented higher food intake and were heavier than MC4R+/+ nonpregnant rats at 15 weeks of age. We then used the MC4R+/− rat to examine the effects of obesity on angiogenic factors, vasorelaxation, and blood pressure phenotypes in pregnancy.

### Angiogenic balance in obese normal pregnant rats

Angiogenic balance is critical for maintenance of healthy pregnancy. This balance is commonly defined by the VEGF (angiogenic):sFlt-1 (antiangiogenic) ratio (Takano et al. 2010). VEGF exerts its proangiogenic actions by promoting endothelial cell proliferation and migration (Ferroni et al. 2012). In vitro, this molecule elicits endothelial tube formation as a precursor to mature blood vessel formation (Miura et al. 2005). During gestation, the rise in maternal VEGF levels is due largely to production in the placenta to meet the angiogenic demands of the utero-placental unit (Wheeler et al. 1999; Geva et al. 2002; Wada et al. 2013). We found under normal pregnant conditions that obese rats have elevated circulating VEGF. However, the placenta was not the origin of the increased VEGF levels in obese pregnant rats, suggesting a maternal source.

White adipose tissue from MC4R+/− obese pregnant rats had greater VEGF and VEGF:sFlt-1 ratio compared to MC4r+/+ lean pregnant rats. Knowledge is lacking about how adipose tissue angiogenic balance influences utero-placental function. Glimpses into adipose tissue angiogenic capabilities have been revealed by studies showing that adipose tissue growth and expansion in obesity depends on angiogenesis (Sun et al. 2011). For example, genetically obese db/db mice have higher circulating and adipose tissue levels of VEGF (Miyazawa-Hoshimoto et al. 2005), and mice with tetracyclin-driven repression of VEGF are resistant to high-fat diet-induced obesity (Lu et al. 2012). Nonpregnant obese women have greater circulating VEGF than nonpregnant lean women (Makey et al. 2013). These data indicate that adipose tissue is a potential reservoir of angiogenic factors, which may contribute to the proangiogenic milieu of pregnancy. Future studies should examine whether VEGF released from the adipose tissue affects maternal and placental vascular function.

Preeclampsia is linked to reduced placental vascularity and perfusion, which is indicated by reduced bioavailable VEGF in the maternal circulation (Kweider et al. 2011). Moreover, placental ischemia in response to reduced uterine perfusion pressure (RUPP) stimulates the placenta to release antiangiogenic factors such as sFlt-1 into the maternal circulation (Gilbert et al. 2007). We and others have also shown that exogenous infusion of sFlt-1 elicits hypertension in normal pregnant rats (Maynard et al. 2003; Karumanchi and Stillman 2006; Murphy et al. 2010). Because soluble factors from the ischemic placenta target and reduce nitric oxide bioavailability, we speculate that the hypertensive and endothelial dysfunction responses to reductions in uterine perfusion may be exaggerated in obese pregnant animals.

### Vasorelaxation in obese normal pregnant rats

We examined how obesity affects the vasorelaxation phenotype in MC4R+/− obese pregnant rats. We found in the obese normal pregnant rats that endothelial dependent vasorelaxation was significantly enhanced in resistance mesenteric arteries over their lean pregnant counterparts. More mechanistically, we found that the response to the exogenous nitric oxide donor sodium nitroprusside was enhanced in the obese pregnant rats. These data indicate that nitric oxide dependent vasorelaxation is enhanced in obese pregnant rats. Previously, we have shown that nitric oxide synthase (NOS) function is enhanced in normal pregnant Sprague Dawley rats, which had a much greater blood pressure response to nonselective NOS inhibition with l-NAME (l-NG-Nitroarginine methyl ester) with respect to virgin controls (Kassab et al. 1998). Future studies will address this in our obese pregnant model.

While our present study focused on the mesenteric circulation, obesity may also affect other important parts of the circulation such as renal and uterine vessels. Studies by Cooke and Davidge (2003) showed that pregnancy exaggerates vasorelaxation similarly in mesenteric and uterine arteries. They observed in wire myography experiments that pregnancy enhanced NOS-mediated vasorelaxation in both arteries. Therefore, we speculate that the enhanced vasorelaxation in our genetically obese pregnant rats is not solely localized to mesenteric arteries.

We propose that blood pressure was not raised in obese pregnant rats due to increased vasorelaxation mediated through a nitric oxide pathway. Although this may be true, it is also important to remember that our obese pregnant rats are heterozygous for the MC4R gene in the whole body. While MC4R-deficient humans are obese, they are not hypertensive (Greenfield 2011). This has also been demonstrated in MC4R+/− and MC4R−/− knockout male mice (Tallam et al. 2005). More mechanistic studies in male Zucker rats have pinpointed that the hypothalamic melanocortin system elicits obesity-induced hypertension (do Carmo et al. 2012). It also seems that reduced brain MC4R protects against loss of systemic NOS function in obesity (do Carmo et al. 2011). These mechanisms may explain why not all obese pregnant women do not develop preeclampsia. Future studies will examine the relative contribution of brain MC4R and systemic nitric oxide pathways on blood pressure regulation in obese pregnant animals.

### Perspectives

Our study assessed cardiovascular outcomes of pregnancy in MC4R-deficient obese rats. This study was conducted under normal pregnant conditions where genetically obese pregnant rats do not present with hypertension. These data are clinically relevant because not all obese pregnant women develop preeclampsia and go on to have normal, healthy pregnancies. We propose that the mechanisms found in our study explain this phenomenon. However, the exaggerated pro-angiogenic and increased vasorelaxation in genetically obese pregnant animals may not be applicable in a preeclamptic setting, such as that found in the RUPP model. We have previously shown that RUPP in Sprague Dawley rats elicits hypertension and angiogenic imbalance with increased circulating levels of sFlt-1 (Gilbert et al. 2007). It has been shown that sFlt-1-mediated antagonism of VEGF sensitizes endothelial cells to pro-inflammatory TNF alpha-induced reductions in nitric oxide signaling (Cindrova-Davies et al. 2011). Intriguingly, circulating TNF alpha was already elevated under normal pregnant conditions in our obese pregnant rats. Therefore, we propose that, because obese MC4R+/− rats have greater dependency on nitric oxide signaling-induced vasorelaxation, genetically obese pregnant animals may have greater blood pressure and vascular dysfunction responses in the face of RUPP due to a rise in placental ischemia-associated factors that target and reduce nitric oxide signaling.
